# Emergence of Two Rare Malignancies, Langerhans Cell Sarcoma and Myeloid Sarcoma, After Targeted Therapy for Chronic Lymphocytic Leukaemia: A Case Report

**DOI:** 10.7759/cureus.114831

**Published:** 2026-08-20

**Authors:** A. Niblock, Niall Mannion, Kerrie Sweeney

**Affiliations:** 1 School of Medicine, University of Ulster, Derry~Londonderry, GBR; 2 Haematology, Antrim Area Hospital, Antrim, GBR

**Keywords:** b-cell lymphoproliferative disorder, case report, cll, langerhans sarcoma, lcs, myeloid sarcoma, pyrexia

## Abstract

Chronic lymphocytic leukaemia (CLL), Langerhans cell sarcoma (LCS), and myeloid sarcoma are distinct haematological malignancies. While CLL is a common mature B-cell lymphoproliferative disorder, LCS and isolated myeloid sarcoma are rare entities that can present significant diagnostic and therapeutic challenges. We report the case of a patient with established CLL whose clinical condition deteriorated rapidly during treatment. Computed tomography (CT) imaging demonstrated the development of new masses that were initially suspicious for Richter's transformation. However, tissue biopsy confirmed the unexpected diagnosis of LCS. Chemotherapy for the LCS was commenced, initially demonstrating a favourable response. Three months later, the patient re-presented with pyrexia and new masses identified on repeat CT imaging. A further biopsy unexpectedly revealed myeloid sarcoma. Despite further investigation and management, the patient's condition continued to deteriorate, and they subsequently died from progressive disease. The sequential development of two exceptionally rare malignancies in a patient with CLL raises important questions regarding disease pathogenesis, clonal evolution, tumour surveillance, and the potential influence of novel targeted therapies. No *BRAF* mutation was detected in either the CLL or LCS biopsy specimens, making it difficult to establish a mutational link between the malignancies. To our knowledge, the occurrence of LCS followed by myeloid sarcoma in a patient with CLL has not previously been reported. This case highlights the importance of repeat tissue biopsy when disease progression does not follow the anticipated clinical course.

## Introduction

Chronic lymphocytic leukaemia (CLL) is a mature B-cell lymphoproliferative disorder characterised by the persistent presence of ≥5 × 10⁹/L clonal B lymphocytes in the peripheral blood for a minimum of three months. Diagnosis is established by flow cytometry demonstrating a characteristic immunophenotype, with co-expression of specific markers such as CD5, CD19, CD20 and CD23 [1]. Studies in most Western countries have demonstrated that CLL is one of the most common haematological malignancies in adults, with an estimated incidence of approximately six in 100,000 people [1,2].

Langerhans cell sarcoma (LCS) is an extremely rare malignant tumour that is capable of local tissue invasion, fast growth and a strong potential to metastasise. LCS is rare, with no more than 100 cases documented in worldwide literature since its classification. The prognosis is very poor, with a mean disease-specific survival of 27 months [3].

Myeloid sarcoma, formerly termed granulocytic sarcoma, is an uncommon tumour that starts outside the bone marrow. Myeloid sarcoma without simultaneous acute myeloid leukaemia (AML) is known as isolated or primary myeloid sarcoma. This is rare, accounting for less than 1% of all AML cases, with an overall incidence of roughly 0.7-2 cases per million adults [4].

Although CLL, LCS, and myeloid sarcoma are recognised as distinct haematological malignancies, the sequential occurrence of all three diseases in a single patient is exceptionally rare, and the biological relationship between them remains poorly understood. Reporting unusual presentations of rare neoplasms is important to improve understanding of disease biology, potential clonal relationships, and the impact of evolving therapeutic strategies on the development of secondary malignancies. This case also underscores the diagnostic value of repeat tissue biopsy in the setting of atypical disease progression, where assumptions based on a pre-existing haematological diagnosis may delay recognition of an alternative pathological process.

## Case presentation

A 71-year-old lady was referred to haematology following four months of widespread lymphadenopathy and significant lethargy. Clinical examination demonstrated widespread palpable cervical, axillary and inguinal lymphadenopathy. No hepatosplenomegaly was detected. Her past medical history included osteoarthritis, hypertension and hypothyroidism. Her brother had passed away aged 61 from pancreatic cancer.

Initial full blood count demonstrated haemoglobin of 110 g/L (reference range, 120-150 g/L), white blood cells of 93.8x10^9^ (reference range, 4.0-10.0x10^9^/L), and significant lymphocytosis of 85.36 x10^9^/L (reference range, 1.0-3.0x10^9^/L). Peripheral blood immunophenotyping confirmed CLL with a CLL clone 5/5 (CD5+, CD19+, FMC7-, CD79-, weak/moderate Ig expression). The patient was negative for the del(17P), *TP53* mutation, and had unmutated *IGHV*.

Computed tomography (CT) scan of the chest, abdomen, and pelvis (CAP) found extensive low-volume lymph node enlargement from the base of the skull to the groin. Subsequent left axilla lymph node core needle biopsy was consistent with small lymphocytic lymphoma (SLL)/CLL phenotype. The patient was classified as having Binet stage B disease, reflecting the presence of extensive lymphadenopathy and absence of significant cytopenias [5].

Given the presence of symptomatic disease, progressive lymphadenopathy, and a lymphocyte doubling time of less than six months, systemic treatment was indicated. The patient was commenced on obinutuzumab and venetoclax, which was well tolerated. This treatment combination elicited a rapid haematological response, resulting in a decrease in lymphocytes to approximately 0.9 x10^9^/L after just two months.

Unfortunately, two months later the patient was admitted to hospital with pyrexia, nausea, vomiting and general aches. Differential diagnosis at this stage was thought likely infection or treatment-related complications. Initially she was treated with IV Tazocin, then later switched to IV meropenem. However, no improvement was noted with antibiotics and the septic screen returned negative. A CT CAP was performed to look for an occult infective source or other malignant cause of her current presentation, such as progressive CLL or transformation to a high-grade B cell lymphoma (Richter's transformation). 

**Figure 1:**
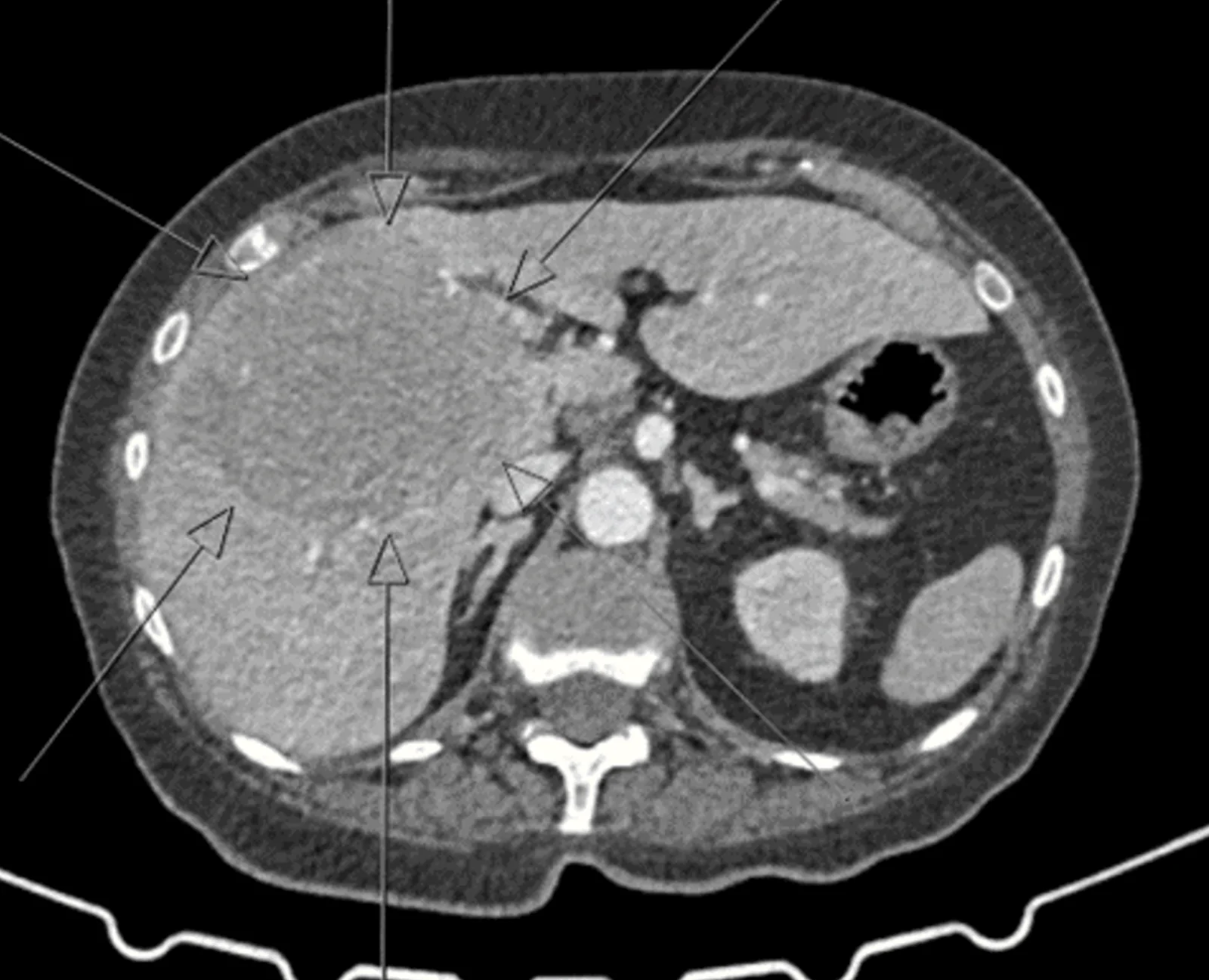
Arterial-phase CT section demonstrating the large mass within the liver Arrows indicate a hypodense hepatic mass lesion

**Figure 2:**
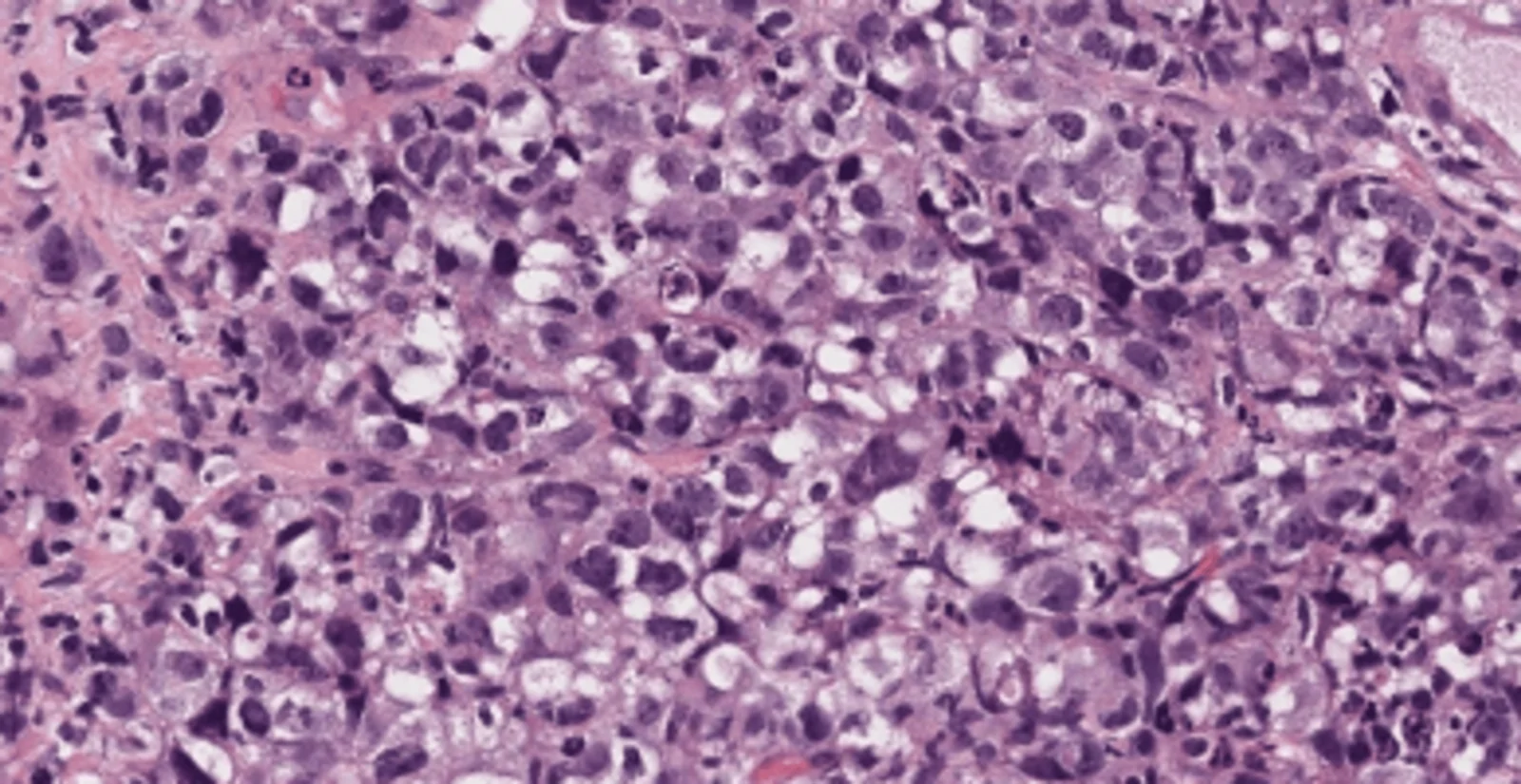
H&E stained core of liver biopsy (x40 with digital zoom) These tumour cells were strongly positive for S100, CD207, CD11c, CD45, CD4 and CD43 but negative for CD163, MPX, Granzyme B, TIA-1 and CXCL13. The features are those of a Langerhans cell sarcoma

CT imaging demonstrated new kidney, liver, and L2 lesions. The hepatic lesion was the largest and most accessible lesion for biopsy (Figure 1). Biopsy was performed and found no evidence of CLL or Richter's transformation. The biopsy was strongly positive for S100, CD207, CD11c, CD45, CD4 and CD43 (Figure 2). Histopathological examination demonstrated a high Ki-67 proliferation index of 70-80%. Epithelial markers (AE1/AE3, CAM 5.2, and BerEP4) were negative. The material was sent to multiple pathologists who agreed this was LCS. Samples were processed for the *BRAF* mutation to look for a connection between the diseases; however was not found to be present.

Following multidisciplinary discussion, treatment with CHOP (cyclophosphamide, doxorubicin, vincristine and prednisolone) chemotherapy was commenced for LCS while obinutuzumab therapy for CLL was continued. Interim CT scanning after three cycles of CHOP showed a good partial response with residual liver mass reducing from 78 mm to 16 mm.

Unfortunately, six weeks later the patient was readmitted with chest pain, cough and pyrexia. All blood cultures were again negative, and treatment included an extensive number of antibiotics with no clinical improvement. Given her previous history, we had a low threshold to repeat the CT imaging, which unfortunately demonstrated an increase in liver lesions. Given the rarity of LCS and the history of CLL, we performed another biopsy. The differential diagnoses at this stage included progression of CLL or LCS, Richter's transformation, or another secondary malignancy.

On this occasion, the biopsy was reported as myeloid sarcoma, based on the overall morphological and immunophenotypic findings. Hepatic core was effaced by a diffuse infiltration of extensive necrosis with areas of ghost cells. Where viable, the cells were positive for some myeloid markers (PU1, CD33, CD13) but did not stain with other monocytic and histiocytic markers. They were CD45+ with no other B (Pax5, CD20, CD79), T-lymphoid markers, IRF4, CD30, or LMP1, and epithelial markers were negative. Reportable variants in the following genes were identified: *BRAF* and *TP53*. A *BRAF* variant was identified of unknown significance. Variants of unknown significance were identified in the following genes: *CBL* and *CSF3R*. Unfortunately, molecular polymerase chain reaction (PCR) failed on the tissue.

Both liver biopsy specimens underwent independent expert pathological review, confirming the diagnoses of LCS and subsequent myeloid sarcoma, respectively. Retrospective analysis demonstrated that the *BRAF* variant identified in the myeloid sarcoma was not present in the preceding CLL or LCS specimens. Despite supportive management, the patient's clinical condition continued to deteriorate. Owing to progressive disease and declining performance status, no further disease-directed therapy was possible, and she died later that month.

**Figure 3:**
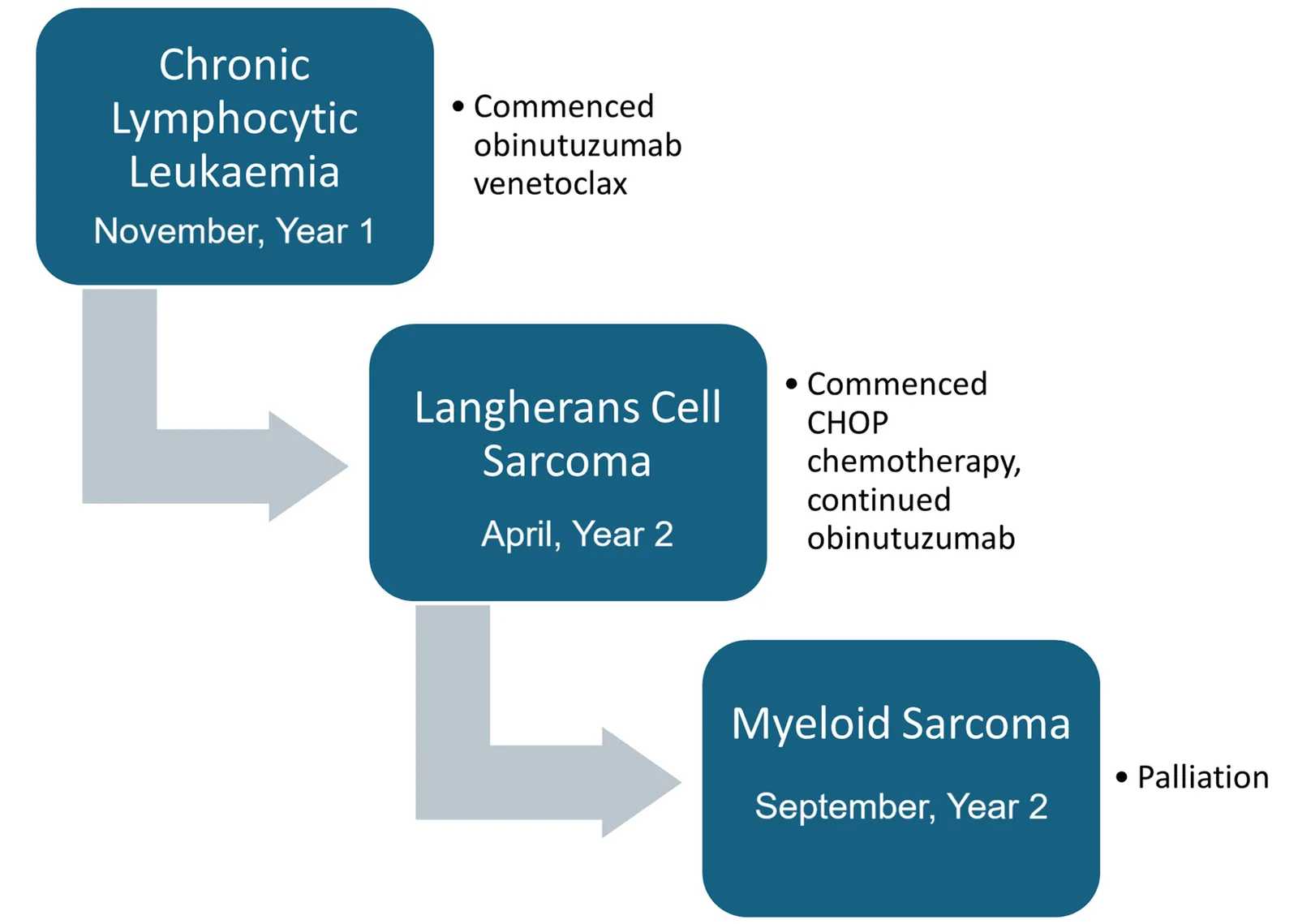
Clinical timeline illustrating the sequential development of CLL, LCS, and myeloid sarcoma CLL: chronic lymphocytic leukaemia; LCS: Langerhans cell sarcoma; CHOP: cyclophosphamide, hydroxydaunorubicin/doxorubicin, Oncovin/vincristine, prednisone/prednisolone

Figure 3 outlines the timeline of each of the sequential diagnoses.

## Discussion

Although outcomes for patients with CLL have improved substantially with the introduction of targeted therapies, the development of secondary malignancies remains an important cause of morbidity and mortality. The increased risk of secondary malignancies in CLL has been attributed to immune dysregulation, impaired tumour surveillance, genetic instability, treatment-related factors, and the underlying disease biology [6]. Impaired immune surveillance has been implicated in the development of both haematological and non-haematological malignancies, although the precise mechanism remains unclear [7]. In the present case, the sequential occurrence of LCS followed by myeloid sarcoma created major diagnostic and therapeutic challenges and represents an exceptionally unusual disease trajectory.

The pathogenesis of LCS remains poorly understood because of its rarity. Reported risk factors include viral infections, immunosuppression, previous haematological malignancies, and exposure to immunosuppressive therapies [3]. Several published cases have described the development of LCS in association with lymphoid neoplasms, particularly CLL/SLL, suggesting a possible biological relationship between these disorders. Previous studies have demonstrated that CLL/SLL can transform into LCS through the process of transdifferentiation [8,9]. Molecular analyses have revealed identical immunoglobulin heavy-chain gene rearrangements in CLL/SLL and subsequent histiocytic or dendritic cell sarcomas, supporting a shared clonal origin and providing strong evidence for transdifferentiation rather than the development of an unrelated second malignancy [9]. More recent studies have identified recurrent mitogen-activated protein kinase (MAPK) pathway alterations and suggest that additional genetic events may be required for lineage conversion and tumour progression [10].

Transdifferentiation refers to the direct conversion of a mature neoplastic cell into another lineage without reversion to a pluripotent precursor state. Research has demonstrated the presence of *BRAF* V600E mutations in some cases of clonally related CLL/SLL and LCS [8]. Although a *BRAF* variant of uncertain significance was subsequently detected in the myeloid sarcoma specimen, the pathogenic *BRAF* V600E mutation was not identified in either the CLL or LCS samples. This makes the biological relationship between the malignancies less certain and suggests that the LCS may have arisen through an alternative pathogenic mechanism. This finding contrasts with several previously reported cases in which molecular evidence supported a direct clonal relationship between the lymphoid and histiocytic neoplasms [9,10].

Merkel cell polyomavirus (MCP) is best known for its association with Merkel cell carcinoma but has also been detected in some cases of LCS, suggesting a potential role in disease pathogenesis [11]. As this patient was not tested for MCP, no conclusions can be drawn regarding its contribution to the development of LCS or subsequent malignancies. Nevertheless, the occurrence of multiple rare sequential haematological neoplasms raises important questions regarding the role of immune dysregulation and tumour surveillance failure. Immune dysfunction is a recognised hallmark of CLL and contributes to an increased risk of infections, secondary neoplasms, autoimmune phenomena, and impaired anti-tumour immune responses [6]. The development of multiple sequential malignancies in this patient may therefore reflect cumulative defects in immune surveillance in addition to disease-specific biological factors.

The subsequent development of myeloid sarcoma following both CLL and LCS is particularly noteworthy. To our knowledge, following a literature search of PubMed and Google Scholar using the terms “chronic lymphocytic leukaemia”, “CLL”, “Langerhans cell sarcoma”, and “myeloid sarcoma”, no previous reports describing the sequential occurrence of CLL, LCS, and MS in the same patient were identified. Myeloid sarcoma most commonly occurs in association with AML and only rarely develops in patients with other haematological malignancies. Reports describing myeloid sarcoma following LCS are exceedingly rare, limiting direct comparison with existing literature. Similar to previously reported cases, diagnosis in our patient required histopathological confirmation because clinical and radiological findings are often non-specific and may mimic progression of an existing malignancy or the development of a solid tumour [12]. This emphasises the importance of maintaining a broad differential diagnosis and obtaining tissue confirmation whenever patients with established haematological malignancies develop new lesions, rapid disease progression, or atypical clinical features.

Overall, this case demonstrates the diagnostic complexity associated with sequential rare haematological neoplasms and highlights the critical importance of repeat tissue sampling when disease behaviour deviates from the expected clinical course. The discordance between the patient's clinical history and evolving pathological findings underscores the need to reassess disease biology throughout the disease trajectory to identify transformation, clonal evolution, or the emergence of a secondary malignancy.

## Conclusions

This case describes the exceptionally rare sequential occurrence of CLL, LCS, and myeloid sarcoma in a single patient. The biological relationship between these malignancies remains uncertain, particularly in the absence of a detectable *BRAF* V600E mutation. The principal learning point is the importance of obtaining tissue diagnosis and performing repeat biopsies when patients demonstrate unexpected clinical deterioration, atypical disease features, or apparent disease progression. Sequential histopathological assessment is essential for identifying transformation, clonal evolution, or the emergence of a second malignancy, all of which may significantly influence treatment decisions and prognosis.
